# Potential confounding in the association between short birth intervals and increased neonatal, infant, and child mortality

**DOI:** 10.3402/gha.v8.29724

**Published:** 2015-11-09

**Authors:** Jamie Perin, Neff Walker

**Affiliations:** 1 Institute for International Programs, Bloomberg School of Public Health, Johns Hopkins University, Baltimore, MD, USA; 2 Department of Pediatrics, Center for Child and Community Health Research, Johns Hopkins School of Medicine, Baltimore, MD, USA

**Keywords:** family planning, fertility, contraception rate, confounding, attributable fraction

## Abstract

**Background:**

Recent steep declines in child mortality have been attributed in part to increased use of contraceptives and the resulting change in fertility behaviour, including an increase in the time between births. Previous observational studies have documented strong associations between short birth spacing and an increase in the risk of neonatal, infant, and under-five mortality, compared to births with longer preceding birth intervals. In this analysis, we compare two methods to estimate the association between short birth intervals and mortality risk to better inform modelling efforts linking family planning and mortality in children.

**Objectives:**

Our goal was to estimate the mortality risk for neonates, infants, and young children by preceding birth space using household survey data, controlling for mother-level factors and to compare the results to those from previous analyses with survey data.

**Design:**

We assessed the potential for confounding when estimating the relative mortality risk by preceding birth interval and estimated mortality risk by birth interval in four categories: less than 18 months, 18–23 months, 24–35 months, and 36 months or longer. We estimated the relative risks among women who were 35 and older at the time of the survey with two methods: in a Cox proportional hazards regression adjusting for potential confounders and also by stratifying Cox regression by mother, to control for all factors that remain constant over a woman's childbearing years. We estimated the overall effects for birth spacing in a meta-analysis with random survey effects.

**Results:**

We identified several factors known for their associations with neonatal, infant, and child mortality that are also associated with preceding birth interval. When estimating the effect of birth spacing on mortality, we found that regression adjustment for these factors does not substantially change the risk ratio for short birth intervals compared to an unadjusted mortality ratio. For birth intervals less than 18 months, standard regression adjustment for confounding factors estimated a risk ratio for neonatal mortality of 2.28 (95% confidence interval: 2.18–2.37). This same effect estimated within mother is 1.57 (95% confidence interval: 1.52–1.63), a decline of almost one-third in the effect on neonatal mortality.

**Conclusions:**

Neonatal, infant, and child mortality are strongly and significantly related to preceding birth interval, where births within a short interval of time after the previous birth have increased mortality. Previous analyses have demonstrated this relationship on average across all births; however, women who have short spaces between births are different from women with long spaces. Among women 35 years and older where a comparison of birth spaces within mother is possible, we find a much reduced although still significant effect of short birth spaces on child mortality.

**Paper context**

Previous analyses have demonstrated increased neonatal, infant, and child mortality with short preceding birth spaces. This relationship has been estimated as an average or adjusted average increased risk across all births; however, women who have short spaces between births tend to be different from women with longer spaces. Among those instances where a comparison of birth spaces within mother was possible, we estimated the effect of short birth spaces on these mortalities and found a reduced, although still significant, effect.

There is an extensive literature linking family planning programmes, contraceptive use, fertility, and the health of infants and young children. Many studies have found a strong association between increasing use of contraceptives or reduced fertility and decreases in child mortality. One explanation of this relationship has been that as women have access to contraceptives, their children are born in different circumstances more favourable for survival (1).

Circumstances at birth conferring excess risk that also tend to decline with increasing contraceptive use have been broadly defined by three categories: age of mother (young and old age), birth parity (first births and higher parity births), and short intervals of time between births or pregnancies (less than 24 or 18 months between births). There has been considerable effort to quantify the risk associated with these factors. In general, analyses to estimate relative risk have proceeded by comparing birth outcomes among various groups with observational data. These different birth conditions have been repeatedly linked to increased poor birth outcomes (e.g. low birth weight, prematurity, small for gestational age) using information from trial results and analysis of facility-based data (2–4).

For most low- and middle-income countries, analyses have generally used national household survey data with full retrospective birth histories [mostly Demographic and Health Surveys (DHS), but also some Multiple Indicator Cluster Surveys (MICS)] to link birth conditions and poor maternal and child health. Surveys have reasonable accuracy when it comes to their recorded birth histories and the real timing of births (5). However, surveys do not generally measure clinical conditions well, and so these analyses primarily link birth risk categories to the risk of mortality, including neonatal, infant, and under-five mortality. For example, mortality in children born to women under 18 has been compared to mortality in children born to women aged 18–34 and shown to be generally higher; mortality in children born to women 35 or older has also been shown to be higher than for children born to mothers aged 18–34 at birth.

The association between these factors and increased child mortality is not necessarily causal or attributable. Associations could be confounded by other factors, such as differences between mothers who have births with these conditions or circumstances that cause both short interbirth intervals and child mortality, such as limited access to health services, including contraception. In addition, an association could also be explained by women being more likely to use contraceptives and reduce fertility when child mortality has dropped (replacement effect) (6).

The relationship between birth spacing and mortality is of interest for determining the attributable fraction of child mortality and predicting the impact of related interventions with the Lives Saved Tool (LiST) (7). Analyses of the association between mortality and birth conditions have often necessarily been conducted with observational data (3). These studies have attempted to address issues of selection and to control for possible confounding. In an analysis with retrospective birth histories from a selection of DHS, Rutstein and Winter (8) used Cox proportional hazards by including additional factors such as mother's education, wealth quintile, and urban or rural residence as predictors in their proportional hazard regressions of neonatal, infant, and child survival (9). DaVanzo and colleagues also used Cox proportional hazards for infant and child mortality and their relationship to interpregnancy intervals, including socio-economic status and other factors as predictors (10). In a recent systematic review, Conde-Agudelo et al. analysed this interpregnancy interval and 57 other similar observational studies examining the relationship between birth spacing and adverse outcomes for maternal, perinatal, and child health. Their inclusion criteria allowed for adjustment for socio-economic status and maternal age, excluding only studies examining the unadjusted relationship (i.e. without any additional predictors) between birth spacing and mortality or other adverse outcome (11). These analyses are not directly comparable, because their definition of short birth interval and adverse outcomes and their sample populations are not necessarily consistent. However, they share the primary statistical methodology of assessing survival with Cox proportional hazards, and they both make adjustments for potential confounders by including additional factors in these regressions. In general, these analyses have not restricted their sample of births or mothers for analysis.

While in theory this approach eliminates some confounding, such adjustment does not necessarily rule out all possible bias due to related factors. First, there may be confounders that have not been measured or have been poorly measured, for example, access to health services or women's empowerment. Second, even confounders that are known and measured may violate model assumptions and lead to biased or imprecise estimates of effect (12).

Recent work by Kozuki and Walker (13) used a different approach to control for selection issues in the analysis of birth spacing. They limited analysis to birth histories from women who were over 35 at the time of survey. Then, within those women who had three or more births, they looked for a short-spaced birth and a regular-spaced birth from the same birth history using conditional logistic regression. The purpose of this approach was to eliminate differences between mothers as each mother provided a short-spaced and regular-spaced birth. In this analysis, there was still an increased risk of mortality for the children born with a short space, however, this risk (OR=1.32) was considerably less than had been reported by other analyses. Is the observed increased mortality risk associated with short birth spacing due to differences between mothers? The analyses presented in this paper seek to extend the work of Kozuki and Walker in assessing the sensitivity of the link between short birth spacing and increased risk of mortality in children.

In this paper, we have two primary purposes. First, we investigate the background differences between women who have short birth intervals and those women who do not. Second, we directly compare the standard cross-sectional analysis of mortality risk to the within-mother analysis.

## Methods

### Data

We used complete birth histories from 145 standard, interim, or continuous DHS household surveys conducted since 1998, including DHS phases III through VI, from 66 unique countries. We included retrospective birth histories from all of these surveys in our analysis of short birth intervals and the women who have children with short birth intervals, while excluding multiple births.

We used these birth histories to assess the potential for confounding in the relationship between short birth intervals and child mortality. To describe women with short birth intervals, we grouped mothers according to their shortest birth interval in four categories (less than 18 months, 18–23 months, 24–35 months, or longer) and summarised the wealth quintile, education, age, and fertility of each group. We also described the observed mortality rates for the children of these women in each category of preceding birth interval.

In addition to assessing the potential for confounding, we also analysed these birth history data to assess mortality risk. For this primary analysis, we also excluded birth histories from women who were younger than 35 when their birth history was recorded, at the time of survey. We limited our analysis to women who were older at survey for several reasons. First, these women are nearing the end of their childbearing years, and so we can estimate their fertility, an additional potential confounder for the effect of birth spacing on mortality (14). Second, we expect women with complete or nearly complete birth histories to have more information about birth spacing, because they have had more time to have children and variety in their interbirth intervals. Although some women who are younger when surveyed have short-spaced births, there is less information about them, because their fertility cannot be estimated and because there are fewer siblings for comparison.

### Statistical analysis

We analysed the relationship between birth spacing and neonatal, infant, and under-five mortality using two approaches. First, we estimated the mortality risk ratio by interbirth interval using a standard regression adjustment for potential confounding factors. We used Cox proportional hazards for mortality (survival) outcomes based on retrospective birth histories, censored at the appropriate age and including mother's education in three categories, along with wealth quintile, partner's education, need for family planning satisfied, completed fertility, and area of residence (urban/rural) as potential confounders in the regression analysis, described in Table 1. Although the DHS include a great wealth of cross-sectional information for recent births, the same information for retrospective births is not available. Facility delivery and skilled birth attendance, for example, are available only for births in the previous 5 years. Other salient factors, like immunisation coverage or mother's HIV status, are not available for birth histories because they are measured at a single point in time. Ideally, we would like to adjust for these factors, if they were available.

**Table 1:** Factors used for adjustment when estimating the effect of birth spacing on neonatal, infant, and child mortality in retrospective birth histories from household survey data

Factor	Description
Wealth quintile	Five categories (poorest, poorer, middle, richer, richest) of a wealth index based on household assets, household construction materials, and water and sanitation facilities
Mother's education	Educational attainment of mother or caretaker, in six categories: no education, incomplete primary, complete primary, incomplete secondary, complete secondary, and higher than complete secondary
Area	Type of place of residence (urban or rural)
Partner's education	Partner's education level in five categories: no education, primary, secondary, higher, and unknown
Family planning need satisfied	Met need for family planning services (yes/no)
Fertility	The total number of children born to a woman during her lifetime
Mother's age	Age of the mother at the time of each birth: 35 and older

In addition to this first approach, we estimated the mortality risk ratio by interbirth interval in a within-mother comparison. We also used Cox proportional hazards for mortality outcomes; however, we stratified by mother to compare mortality only for women who had variety in their interbirth spacing. This second analysis was similar to conditional logistic regression, where mortality outcomes are compared for different outcomes within woman, except that conditional logistic regression estimates odds ratios, while Cox regression estimates mortality rate ratios (9, 15). We used Cox regression to estimate relative neonatal, infant, and under-five mortality.

Stratifying proportional hazards regression matches births within woman and so strictly controls for all factors that are constant for mothers over their childbearing years (16). There is still potential for confounding in factors that change over time, for example, mother's age or access to care. It is also possible that education, area of residence, or socio-economic indicators may change over time. We analysed births only for women whose full birth history was available up to age 35, and so births to women who were younger than 35 at the time of survey are excluded. This disproportionately excludes recent births and births of low parity.

We estimated the effect of birth spacing separately for each survey using these two methods. We then combined these results from multiple surveys in a meta-analysis with random survey effects, weighted by the estimated standard error of the birth spacing effect for each survey, for an overall effect estimate for each method. In addition, we assessed the potential for confounding in the within-mother analysis by examining the association between birth spacing and parity. We also described those births that did not contribute to the second within-mother analysis.

## Results

### Differences between mothers and births

Table 2 describes age, average fertility, socio-economic status, and education of mothers in all 145 surveys classified by their shortest birth interval. Birth interval appears to be correlated with factors that are also associated with child mortality.

**Table 2:** Characteristics of mothers classified by their smallest observed birth interval, as raw averages across 145 surveys in 66 countries since 1998, among women who were at least 35 years of age when surveyed

Smallest birth interval	Total number of women (thousands)	Mean (SD) age at survey	Mean (SD) fertility	% in bottom wealth quintile^a^	% with no education
Only first births	45	40.5 (0.6)	1 (na)	12.7 (6.8)	28.9 (26.0)
<18 months	205	41.7 (0.6)	6.5 (1.3)	22.7 (3.7)	41.6 (30.6)
18–23 months	151	41.1 (0.6)	5.6 (1.1)	21.1 (4.2)	38.8 (30.6)
24–35 months	134	41.0 (0.6)	4.4 (0.8)	17.5 (4.0)	33.8 (29.2)
≥36 months	113	40.6 (0.7)	2.8 (0.3)	12.3 (5.9)	28.5 (26.8)

a Wealth quintile data do not include 1999 survey in Nigeria; na, not applicable.

These analyses reveal the differences that exist between mothers who have short-spaced births compared to those who do not. In general, the women who had at least one birth that came 18 months or less after a preceding birth had lower education, were more often from the poorest wealth quintile, and had higher levels of fertility. These factors, and perhaps others that are unmeasured, have the potential to confound the association between shorter birth intervals and risk of mortality in the children of these women.

Table 3 describes births for each chosen category of preceding birth interval. There are over 5 million births recorded by these surveys. Gender and parity do not appear related to birth interval; however, mortality for short intervals is higher than regular or long intervals, as expected.

**Table 3:** Characteristics of births by preceding birth interval in four categories, across 145 DHS since 1998, among women who were at least 35 years of age when surveyed

	Total number of births (thousands)	% (SD) male	Average (SD) birth order	Average under-five mortality rate per 1,000 live births (SD)
First births	645	51.5 (1.4)	1 (na)	128.9 (68.8)
Birth interval				
<18 months	332	51.4 (1.7)	4.2 (0.7)	202.2 (83.6)
18–23 months	406	51.1 (1.6)	4.2 (0.6)	142.4 (71.3)
24–35 months	789	51.0 (1.1)	4.2 (0.6)	108.8 (58.1)
≥36 months	859	50.9 (1.2)	4.2 (0.7)	68.5 (36.6)

Multiple births are excluded; DHS, Demographic and Health Surveys; na, not applicable.

### Method comparison

**Fig. 1:**
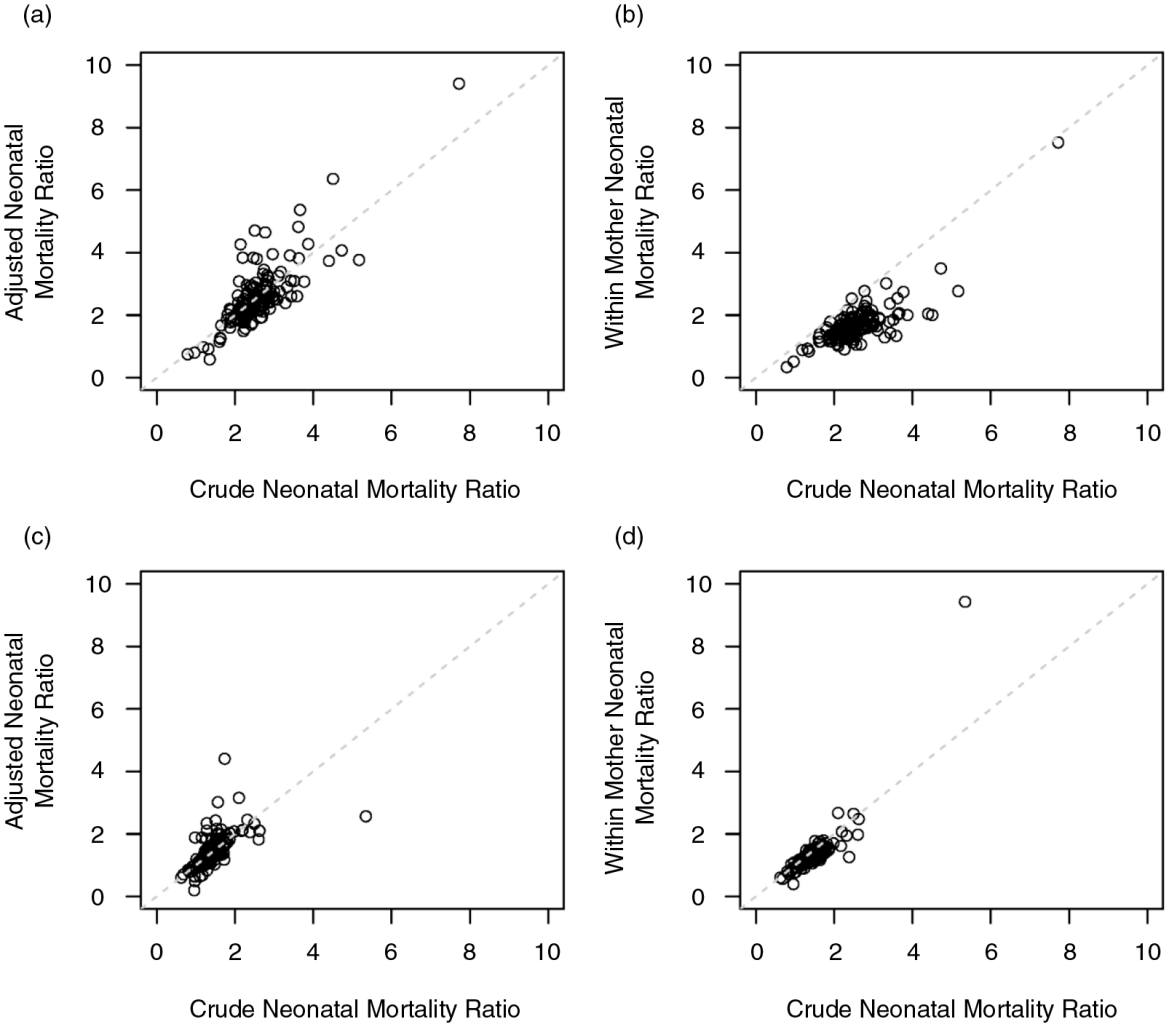
Comparison of crude neonatal mortality rates with those estimated by a standard regression adjustment and by stratifying within mothers. Neonatal mortality ratios are shown for comparing births with preceding interval less than 18 months to 24–35 months [(a) standard regression and (b) stratified regression] and for comparing births with a preceding interval of 18–23 months to those with a 24–35-month interval [(c) standard regression and (d) stratified regression].

We first compared the crude mortality ratios to the adjusted ratios for both the standard regression adjustment approach and the within-mother analysis using the stratified Cox proportional hazards regression. As can be seen in Fig. 1a, standard regression-adjusted estimates are similar to the crude neonatal mortality ratios when comparing births with spacing under 18 months to births with spacing of 24–35 months. However, in Fig. 1b, stratified Cox proportional hazards regression for neonatal mortality estimated a smaller effect of short intervals compared to the crude neonatal mortality ratios. Mortality risk ratios for a preceding birth interval of 18–23 months compared to 24–36 months are also shown in Fig. 1c and d. The results for these slightly larger birth intervals are less different than those for intervals less than 18 months, although there still appears to be a lower mortality ratio using the within-mother comparison.

**Fig. 2:**
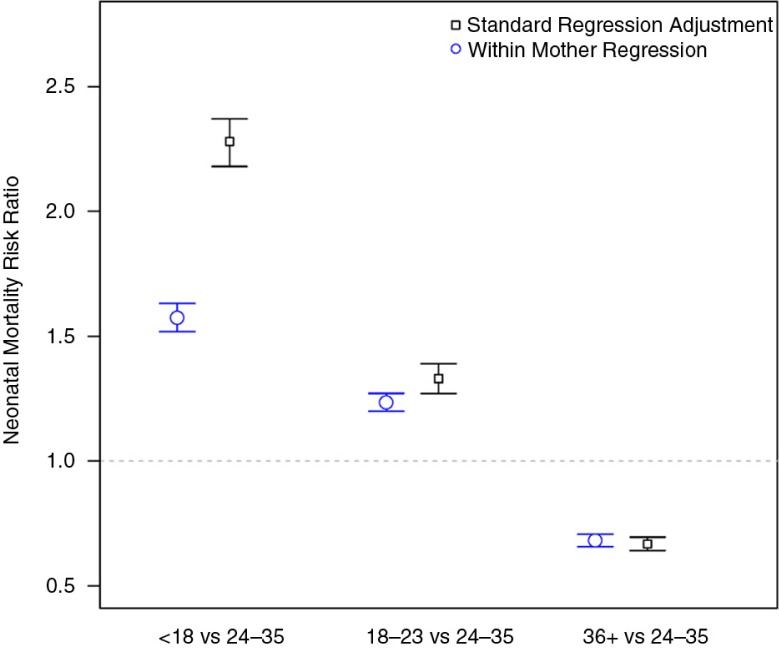
Results of the meta-analysis for the effect of birth spacing on neonatal mortality across 145 DHS, using a standard adjustment for a Cox proportional hazard regression, and another Cox regression estimating the effect of birth spacing on neonatal mortality within mother.

In Fig. 2, we present the overall estimated risk ratios for neonatal mortality for children born with a preceding birth interval under 18 months and between 18 and 24 months as compared to a birth interval of between 24 and 35 months across 145 DHS. We do this separately for both the standard adjusted regression model and the within-mother Cox proportional hazards model. As would be expected based on the results shown in Fig. 1, the within-mother comparisons provided smaller estimates of the risk ratios for neonatal mortality than the adjusted regression analyses. For the shortest interbirth interval (less than 18 months), the additional mortality risk was reduced by almost one-third (2.27, 95% confidence interval: 2.18–2.37 vs. 1.57, 95% confidence interval: 1.52–1.58) using the within-mother technique. The estimated effects of birth interval for neonatal, infant, and child mortality are shown in Table 4 for both the standard adjustment and within-mother methods. Our interpretation is that the risk ratio estimated within mother is a more accurate measure of the direct relationship between birth intervals and mortality than the risk ratio estimated by the adjusted regression for women over 35 years, because births to the same women have more in common than can be specified by what was available in these cross-sectional survey data.

**Table 4:** Average neonatal, infant, and under-five mortality risk ratios by preceding birth interval with 95% confidence limits, across 145 DHS since 1998, for women who were at least 35 at the time of survey

Birth interval	Standard regression adjustment^a^	Cox regression stratified by mother
Neonatal mortality		
<18 months	2.28 (2.18, 2.37)	1.57 (1.52, 1.63)
18–23 months	1.33 (1.27, 1.39)	1.24 (1.20, 0.27)
24–35 months	(Reference)	(Reference)
≥36 months	0.67 (0.64, 0.70)	0.68 (0.66, 0.71)
Infant mortality		
<18 months	2.31 (2.23, 2.39)	1.53 (1.49, 1.58)
18–23 months	1.36 (1.32, 1.41)	1.21 (1.19, 1.24)
24–35 months	(Reference)	(Reference)
≥36 months	0.62 (0.60, 0.64)	0.68 (0.66, 0.70)
Under-five mortality		
<18 months	1.81 (1.75, 1.88)	1.41 (1.37, 1.46)
18–23 months	1.25 (1.22, 1.28)	1.17 (1.15, 1.19)
24–35 months	(Reference)	(Reference)
≥36 months	0.75 (0.73, 0.77)	0.73 (0.71, 0.75)

a Adjusted for wealth quintile, mother's education, area (urban or rural), partner's education, family planning need satisfied, fertility, and mother's age. Birth spacing effects were estimated by Cox proportional hazard regression and separately by another Cox regression estimating the effect of birth spacing on neonatal mortality within mother. DHS, Demographic and Health Surveys.

### Investigating possible selection issues of the within-mother comparisons

Matching births within-mother controls completely for factors that are constant over a woman's childbearing years. Education and socio-economic status, for example, would not be expected to change substantially during this time. Other factors identified for their relationship with child mortality are known to vary significantly during this time period.

**Fig. 3:**
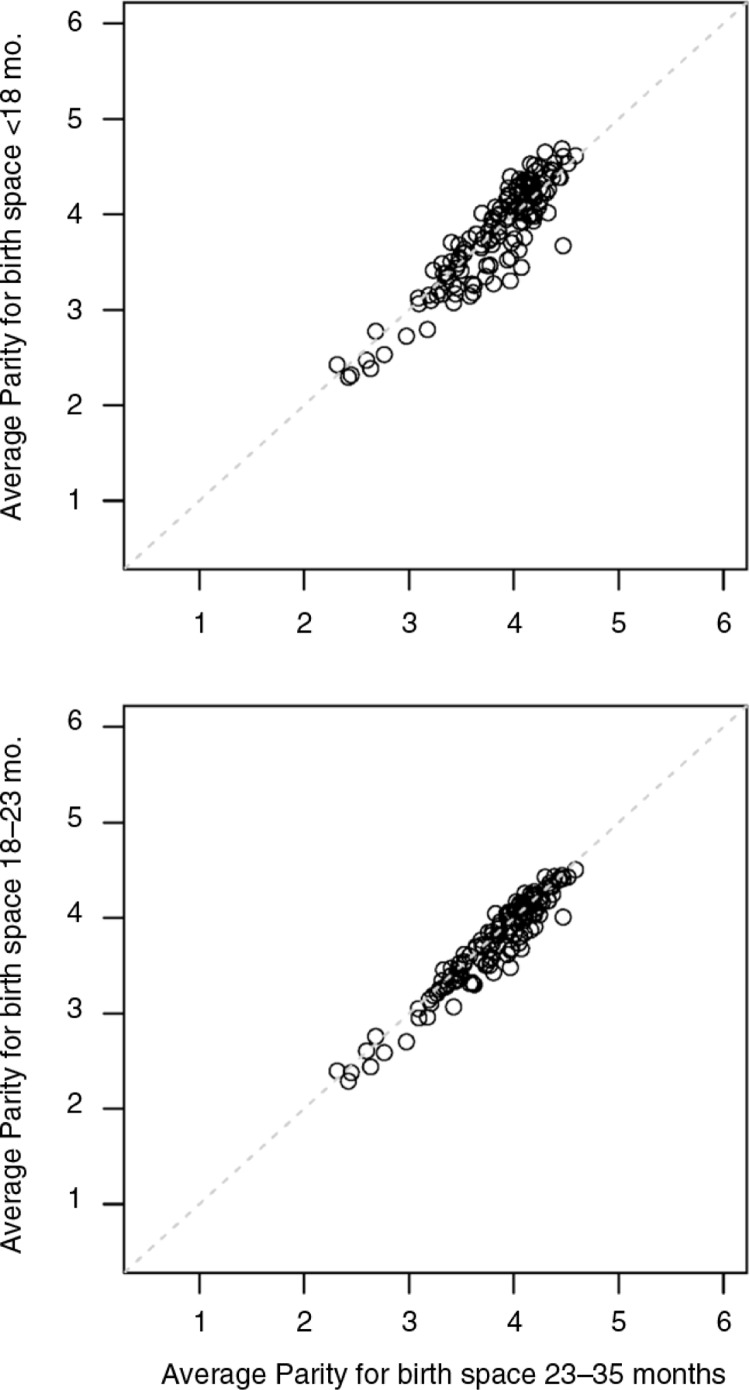
Average parity by survey and for three categories of preceding birth space, for 145 household surveys in 66 countries.

There are two possible selection issues that might make the within-mother comparison biased. First, if there is a difference in parity between the short-spaced and regular-spaced births, it could confound the effect of short birth spacing with the association of mortality and parity. To test this possibility, we computed the average parity for each birth in the within-mother analysis for the various interbirth intervals. Not surprisingly, average parity among all births is highly variable across the DHS in this analysis. However, the difference between average parity of short-spaced births and the average parity of regular-spaced births is very small. The average parity of births with preceding space of 24–35 months was 3.76 (range: 2.3–4.6), while the average parity for births with less than 18 months preceding space is 3.82 (range: 2.3–4.7). The average parity for births with 18–24 months preceding space is 3.76 (range: 2.3–4.5). Scatter plots of these average birth orders are shown in Fig. 3. These findings rule out parity differences as an explanation of the reduced risk ratio found with the within-mother comparison.

A second possible limitation of the within-mother comparison is that requiring mothers to have both a short- and a regular-spaced birth to contribute to the analysis might result in a biased sample of short-spaced births. This would occur because matching births with short spaces to births with optimal space within mother may exclude some births from analysis, since short-spaced births with no optimally spaced sibling have no potential match and do not contribute to the estimated effect of short birth spacing on mortality. This scenario inevitably arises for some women, for example, women with only two births. Such a woman can only have one birth with an interbirth interval, because first births are not subject to a birth space.

In order to assess this possible source of bias, we compared the total number of short-spaced births among women who were at least 35 years old at survey to the number that were used in the within-mother comparison. Of 2.4 million births to women surveyed at 35+years, there were 0.74 million with a preceding space less than 24 months. Overall, 0.69 million (93%) of these births had siblings who were not subject to a short preceding birth interval, and so were matched within-mother and contribute to analysis.

**Fig. 4:**
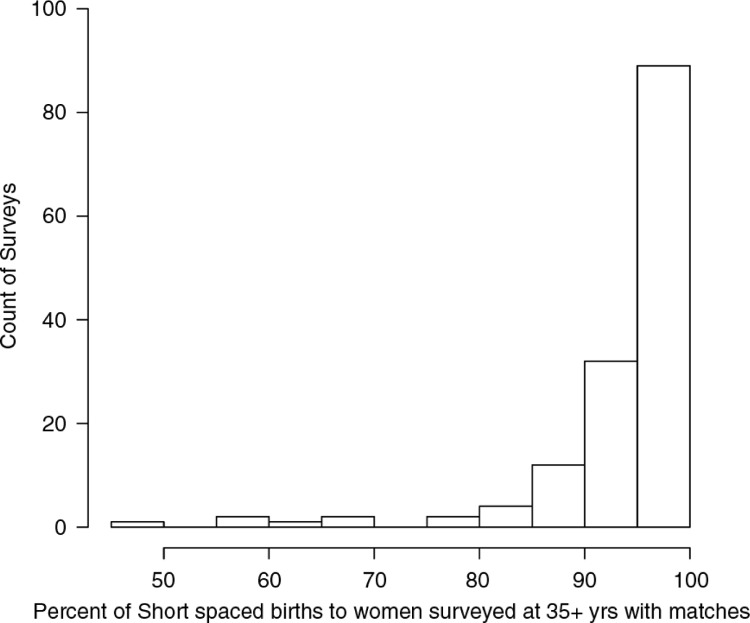
Percentage of short-spaced births that could be matched to another birth for the same mother, in the Cox regression analysis stratified by mother. This percentage is shown for each of 145 household surveys.

While overall the percent of births lost due to matching within mother is low, some surveys were more likely to exclude births than others. The 2007 Ukraine Survey, for example, has the lowest percent of matched short-spaced births (45%), so that more than half of all short-spaced births to women surveyed at 35+ years from this survey were not contributing to analysis. Figure 4 shows the distribution of the percent of short-spaced births with eligible matches for all 145 surveys in our analysis.

This analysis suggests that overall the within-mother technique seems to provide a strong representation of short-spaced births to women who were 35 or older at the time of survey. The vast majority of surveys found that over 90% of short-spaced births could be matched with a regular-spaced birth. The one limitation is that for countries with low fertility such as Ukraine, the percent of matches is reduced and here the risk ratios estimated by the within-mother comparison may not be representative of all short-spaced births to women surveyed at 35 or older.

## Discussion

We estimated the effect of preceding birth interval in four categories on the risk of neonatal, infant, and under-five mortality with recent household survey data from 66 countries. We found ample evidence of a potential for confounding factors when estimating the relationship between birth spacing and mortality in these survey data. We then estimated the relative neonatal, infant, and under-five mortality risks based on two different methods: first, to replicate how this relationship has been determined historically, by including available factors in a proportional hazards regression and, second, with mothers as a unit of stratification among women 35 years and older at survey. Our assumption was that using mothers as strata would be a more efficient method to control for confounders, and the resulting effect estimate would be closer to a direct effect, since this analysis controls for all factors that are constant over a woman's childbearing years. Both this and the former regression adjustment method clearly indicated a statistically significant effect of birth spacing on child mortality. However, the effect estimated by the within-mother analysis was approximately 30% smaller than that estimated with the adjusted regression for neonatal mortality, strongly indicating that there is confounding in the regression-adjusted estimate. Rutstein and Winter (8) used methods similar to this standard adjustment to estimate relative mortality rates by birth-to-conception intervals, with an overall risk ratio for under-five mortality of 3.24 (95% confidence interval: 3.12–3.36) for a birth-to-conception interval less than 6 months and 2.33 (95% confidence interval: 2.25–2.42) for an interval of 6–11 months (8). Although the intervals used in our analysis are not exactly comparable, as we used interbirth time among women aged 35 at survey, we estimate the under-five mortality risk ratio for an interbirth interval less than 18 months to be 1.41 (95% confidence interval: 1.37–1.46).

We found a similar effect for our restricted sample with a standard adjustment method (2.28 for an interbirth interval of 18 months) to that found in a similar unrestricted analysis by Rutstein and Winter (8) (2.33 for a birth-to-conception interval of 6–11 months). The within-mother analysis effectively controls for some factors, although there is still potential for confounding by factors that change for individual women over time. There is no evidence among these survey data, however, that parity is different for different categories of birth spacing, and so parity has little to no potential to confound the effect of birth spacing on mortality.

## Limitations

We restricted our sample to women who were older at the time of survey, and so some births to young women were excluded. In addition, we were not able to include all short-spaced births in the within-mother analysis, because even among women providing birth histories at 35 years or older, not all births had appropriate matches, that is, births by the same mother that were not short-spaced. This was especially true for surveys or countries with low fertility rates. However, we expect that countries with low fertility are also in general countries with lower neonatal and child mortality. Thus excluding births primarily from low-fertility areas may be biased away from the null to estimate an effect larger than the direct effect. In addition, the overwhelming majority of surveys contributed at least 90% of all short-spaced births reported in the birth histories from older women. Finally, due to the matching and restriction of birth history information to older women, we were unable to generalise our results to the entire sample of interest.

## Conclusions

An estimate of the causal relation between birth spacing and neonatal, infant, and under-five mortality is of interest for determining the attributable fraction of child mortality and predicting the impact of interventions with the LiST software. A strong and statistically significant effect has consistently been identified of short preceding spaces on increased child mortality. Using matching and restriction, our study indicates an approximately one-third reduction in the risk of neonatal mortality. We present an alternative comparison of preceding birth spaces within women 35 years and older to estimate a still significant but less strong effect.
